# Fully Autonomous Real-Time Defect Detection for Power Distribution Towers: A Small Target Defect Detection Method Based on YOLOv11n

**DOI:** 10.3390/s25206445

**Published:** 2025-10-18

**Authors:** Jingtao Zhang, Siwen Chen, Wei Wang, Qi Wang

**Affiliations:** 1 Department of Robotics Engineering, Nanjing University of Information Science and Technology, 219 Ningliu Road, Pukou District, Nanjing 210044, China; 202312490059@nuist.edu.cn (J.Z.); 202312220004@nuist.edu.cn (S.C.); 2 Fukushima Institute for Research, Education and Innovation, 6-1 Yazawa-machi, Gongendo, Namie Town, Futaba County, Fukushima 979-1521, Japan; wang.wei.r4k@research.f-rei.go.jp; 3 School of Electronic Engineering, Nanjing Xiaozhuang University, 3601 Hongjing Avenue, Jiangning District, Nanjing 211171, China

**Keywords:** BiFPN, SPD-Conv, CBAM, autonomous flight defect detection, YOLOv11

## Abstract

Drones offer a promising solution for automating distribution tower inspection, but real-time defect detection remains challenging due to limited computational resources and the small size of critical defects. This paper proposes TDD-YOLO, an optimized model based on YOLOv11n, which enhances small defect detection through four key improvements: (1) SPD-Conv preserves fine-grained details, (2) CBAM amplifies defect salience, (3) BiFPN enables efficient multi-scale fusion, and (4) a dedicated high-resolution detection head improves localization precision. Evaluated on a custom dataset, TDD-YOLO achieves an mAP@0.5 of 0.873, outperforming the baseline by 3.9%. When deployed on a Jetson Orin Nano at 640 × 640 resolution, the system achieves an average frame rate of 28 FPS, demonstrating its practical viability for real-time autonomous inspection.

## 1. Introduction

As the energy artery sustaining societal operations, the safe and stable functioning of power distribution systems is critically linked to societal development [1,2]. Within this infrastructure, distribution towers and their components, primarily insulators for electrical insulation and tension clamps for securing conductors, are paramount for reliable power delivery [3,4,5]. However, these components are persistently threatened by a spectrum of defects induced by environmental stressors and operational wear. Beyond the non-standard insulator binding and missing tension clamp shells previously mentioned, common critical failures include insulator cracking or explosion caused by flashovers, corrosion-induced weakening of metal fittings, and the loosening of vital fasteners like bolts and split pins [6]. Although visually minor, these defects can precipitate catastrophic consequences, including localized power outages, large-scale cascading grid failures, and even wildfires, posing significant risks to public safety and economic activity. Consequently, proactively identifying these defects is not merely a maintenance task but a crucial requirement for ensuring the security and efficiency of the entire power system. This study focuses specifically on the automated detection of non-standard insulator binding and missing protective shells on tension clamps, two prevalent yet exceptionally challenging defects due to their small size and low contrast against complex backgrounds.

In recent years, intelligent inspection technologies such as robots [7], helicopters [8], and unmanned aerial vehicles (UAVs) [9,10,11] have been introduced for overcoming the limitations of manual inspection. Among them, UAVs are widely adopted for their flexibility and ability to operate in complex terrains [12], but the acquired images still rely on manual analysis, which is demanding, inefficient, and more likely lead to error, especially for small defects like non-standard insulator bindings and missing tension clamp shells [13,14].

To solve this problem, people have applied machine learning to tower recognition. Initial recognition approaches primarily utilized manually designed feature extraction methods, including Haar-like features and Histogram of Oriented Gradients (HOG), combined with traditional classifiers [15,16,17,18]. However, these approaches suffer from low accuracy, limited efficiency, and poor generalization, making them unsuitable for detecting small defects in large-scale image datasets. Driven by deep learning advancements, methods such as Region-based Convolutional Neural Networks (R-CNNs) and their improved versions improve accuracy but remain heavy for embedded deployment [19,20,21].

However, deployment on edge devices is difficult because computational resources are limited since the two-stage design of these models leads to increased model complexity. To accelerate detection process, Redmon, J. et al. [22] introduced the YOLO algorithm, which significantly improved detection speed while maintaining high accuracy. Subsequent versions of YOLO [23,24,25] have continually enhanced both precision and efficiency.

In recent years, numerous research teams have focused on insulator detection in large-scale high-voltage transmission towers by improving YOLO-based algorithms, achieving significant advancements in both recognition speed and accuracy. Zhiqiang Xing et al. developed an improved model named MobileNet-YOLOv4, making dataset enhancement through Gaussian filtering, K-means clustering, and Mosaic, achieving a recognition accuracy of 97.78% [26]. Nan Zhang et al. introduced Insulator-YOLO, an improved YOLOv5 framework, which enhanced insulator recognition precision with a mean average precision (mAP) of 89.65% [27]. Meng Wang et al. achieved an accuracy of 92% built upon the YOLOv7 architecture by integrating Transformer algorithms, triple attention, and smooth intersection-over-union (IoU) loss algorithms [28]. Guoguang Tan et al. developed the Lightweight-YOLOv8n model through incorporating SimAM attention mechanism and modifying the backbone network, achieving an mAP of 90.6% [29]. Junmei Zhao et al. proposed an improved YOLOv11n model with ODConv convolution and Slimneck, maintaining low computational complexity while achieving a high mAP of 91% [30].

Another group of studies has focused on detecting small defects in power fittings of transmission towers using YOLO-based frameworks, achieving notable improvements in detection precision and speed. Lincong Peng et al. proposed the YOLOv7-CWFD model tailored for bolt defect detection, achieving an mAP of 92.9% [31]. Peng Wang et al. introduced PMW-YOLOv8, an improved object recognition algorithm based on YOLOv8, specifically designed to solve split pin loosening detection [32]. Even when target defects occupied only 0.25% of the total image area, the model attained an mAP of 66.3%. Siyu Xiang et al. developed a modified YOLOv8 architecture to tackle challenges in grading ring defect detection caused by structural similarities between defective and normal components [33]. Their method achieved a 6.8% enhancement in accuracy over the baseline model while minimizing critical defect information loss during feature fusion. Additionally, Zhiwei Jia et al. [34] proposed a live-line detection system for tension clamps in transmission lines using dual UAV collaboration. While the dual-algorithm system achieved a mean precision of 82.7%, it required two UAVs and two recognition models, leading to excessive hardware resource consumption. However, it requires two aircraft and two recognition models, which is too complex and wastes hardware resources, and the accuracy of the improved YOLOv8-TR algorithm is within 60.9%.

The newest research has demonstrated the potential of structurally modifying YOLO architectures for enhanced small-object detection. For instance, Benjumea, A. et al. [35] introduced a series of models, YOLO-Z, by altering components and connections in YOLOv5, achieving a significant mAP gain for small objects with minimal latency cost. In a complementary approach focused on drones, Chen, D. et al. [36] proposed SL-YOLO, which incorporates a dedicated cross-scale fusion network (HEPAN) and lightweight modules to simultaneously improve accuracy and reduce computational complexity on the VisDrone dataset.

A comprehensive analysis of existing YOLO-based improvements for power equipment inspection reveals that their strategies, while effective in their own contexts, fail to address the interconnected challenges of distribution tower defect detection with a holistic and systematic design. The core of the problem lies in a cascade of model limitations when facing minute, low-contrast defects:

- Early-Stage Feature Destruction: Standard convolutional and down-sampling operations irreversibly discard the fine-grained spatial details of tiny defects, leading to an insurmountable information loss at the very beginning of the network.
- Lack of Discriminative Focus: In complex aerial backgrounds, the model’s receptive field is cluttered with irrelevant information, lacking a mechanism to actively suppress background noise and amplify the subtle, defining features of the target defects.
- Inefficient Information Flow: The pathways for fusing shallow, high-resolution features (which contain location details) with deep, semantic features (which provide context) are suboptimal, creating a bottleneck that hinders the construction of robust, multi-scale representations.
- Inadequate Output Resolution for Small Targets: The standard detection heads are often insufficiently sensitive to the sparse and weak feature responses of the smallest defects, limiting the final detection performance.

While prior works have touched upon isolated aspects, such as adding attention modules or modifying feature pyramids, none have proposed a coherent architectural solution that sequentially resolves this entire cascade of challenges. Our work introduces TDD-YOLO as such a solution, with each component meticulously engineered to address a specific link in this problem chain.

To bridge this gap, we propose a tower defect detection framework built upon YOLOv11n (TDD-YOLO). Its architecture is founded on a systematic, four-stage enhancement strategy that guides the visual information from input to output with maximal fidelity and discriminative power:

- Feature Preservation with SPD-Conv: In the backbone network, we replace standard down-sampling layers with Spatial-to-Depth Convolution (SPD-Conv) [37]. This is the foundational step to solve the problem of early-stage feature loss. By converting spatial maps into depth channels without discarding any pixels, SPD-Conv ensures that the fine-grained details of tiny defects are preserved and carried forward into the deeper layers of the network.
- Salience Enhancement with CBAM: Following the feature-preserving convolutions, we integrate the Convolutional Block Attention Module (CBAM) [38]. This module directly addresses the issue of complex backgrounds and insufficient focus. It sequentially applies channel and spatial attention to dynamically weight the feature maps, effectively teaching the model to ’look at’ the defect regions while ignoring irrelevant clutter.
- Efficient Information Fusion with BiFPN: To tackle the inefficiency in multi-scale information propagation, we redesign the neck network using a Bidirectional Feature Pyramid Network (BiFPN) [39]. BiFPN establishes fast, bidirectional cross-scale connections, enabling the high-resolution, detail-rich features and the semantically strong contextual features to be continuously and efficiently fused. This process ensures that the final feature pyramids are rich in both detail and context.
- Precision Output with a High-Resolution Detection Head: Finally, we introduce a dedicated detection head that operates on the highest-resolution feature map. This head is specifically designed to enhance the model’s capability to capture the features of small targets, providing a direct and optimized pathway for locating the minute defects that are easily missed by standard heads.

In essence, TDD-YOLO is not a mere assembly of advanced modules, but a coherent pipeline where each innovation tackles a critical bottleneck, and their sequential integration creates a synergistic effect that is greater than the sum of its parts.

The main contributions of this paper are as follows:

1. We propose a novel, four-stage architectural improvement strategy for small defect detection. This strategy systematically addresses the core challenges of feature loss (via SPD-Conv), lack of focus (via CBAM), inefficient fusion (via BiFPN), and insufficient output sensitivity (via a high-resolution head).
2. We instantiate this strategy in TDD-YOLO, a task-specific model that demonstrates the synergistic effectiveness of the proposed cohesive pipeline on the challenging task of distribution tower defect inspection.
3. We validate the generality of our improvement strategy by successfully applying it to YOLOv8n, demonstrating its consistent performance boost across different base frameworks.
4. We implement a fully autonomous UAV-based inspection system, proving the end-to-end practicality of our model in real-world scenarios.

## 2. Materials and Methods

### 2.1. Model Architecture and Modifications

**Figure 1:**
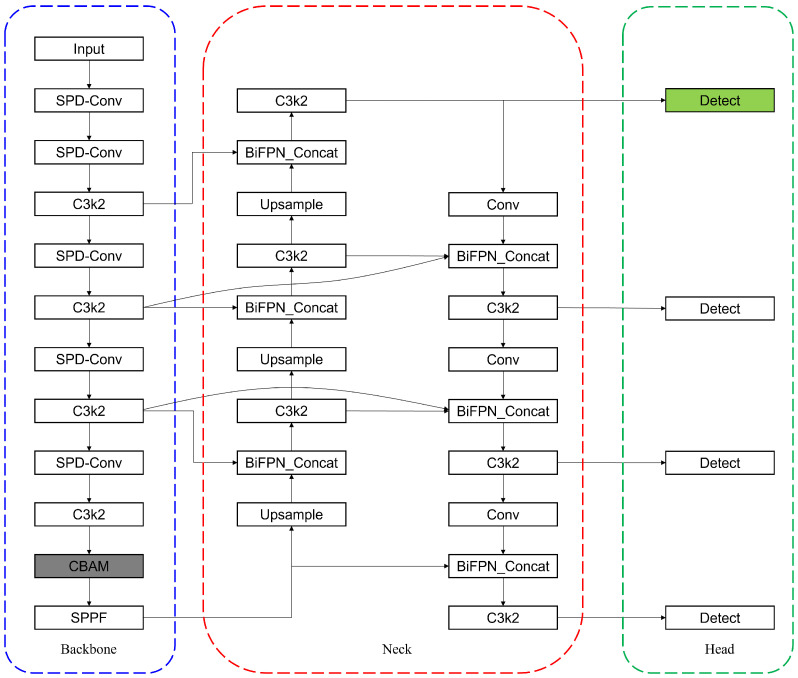
Thearchitecture diagram denotes the key enhancements to the YOLOv11n model through colored dashed boxes and color-highlighted components: the SPD-Conv module (blue), a redesigned BiFPN network (red), a CBAM attention module (gray), and a specialized detection head (green).

Building upon the YOLOv11n architecture, which balances efficiency and performance for edge deployment, we constructed our task-specific model, TDD-YOLO. The overall architecture is designed to implement the synergistic improvement strategy outlined in the introduction, and its complete structure is depicted in Figure 1.

Based on YOLOv11n, the convolution strategy in the backbone was enhanced by replacing standard Conv layers with SPD-Conv, as visually indicated by the blue box in the diagram. The feature extraction network in the Neck section was re-engineered to strengthen multi-scale feature fusion using a BiFPN structure, as marked by the red box in the diagram. The model integrates a CBAM attention module, which is placed before the SPPF module, with the corresponding components marked by gray-shaded modules in the architecture diagram. Additionally, a specialized detection head was incorporated into the Head structure, with the corresponding components highlighted in green in the architecture diagram.

Detailed specifications of the model architecture, including input/output channels and spatial dimensions at each layer, are provided in Table A1.

#### 2.1.1. SPD-Conv Convolution Module

Detecting small objects is still a challenging work by reason of some factors. The primary limitation lies in feature degradation caused by downsampling operations such as strided convolution and pooling. Large objects retain sufficient feature representation during downsampling due to their broad spatial coverage and rich texture. In contrast, small objects, with their limited pixel area and inherently low resolution, suffer significant information loss during downsampling, thus increasing the likelihood of false negatives or erroneous detection.

To tackle these challenges, this study take the SPD-Conv in place of the traditional strided convolution in YOLOv11n. SPD-Conv is a novel component that integrates a Space-to-Depth (SPD) transformation with standard convolution. This operation transfers the spatial dimensions of feature maps into channel dimensions, effectively preserving spatial information during downsampling.

In addition, the innovation of the SPD-Conv lies in its space-to-depth transformation. This operation transfers the spatial dimensions of the input feature map into the channel dimension, effectively preserving spatial information during downsampling.

Given an input feature map of size S×S with $C_{1}$ channels, the transformation begins with non-strided convolution layers that expand the channel dimension to $C_{2}$ while maintaining the original spatial resolution S×S. Subsequently, the core space-to-depth operation applies a scaling factor *s* (typically s=2) to partition the spatial domain. This decomposition splits the feature map into $s^{2}$ spatial sub-windows, each of size $\left(S/s\right)\times \left(S/s\right)\times C_{2}$. These sub-maps are then concatenated along the channel dimension, producing an intermediate representation of size $\left(S/s\right)\times \left(S/s\right)\times \left(s^{2}C_{2}\right)$.

**Figure 2:**
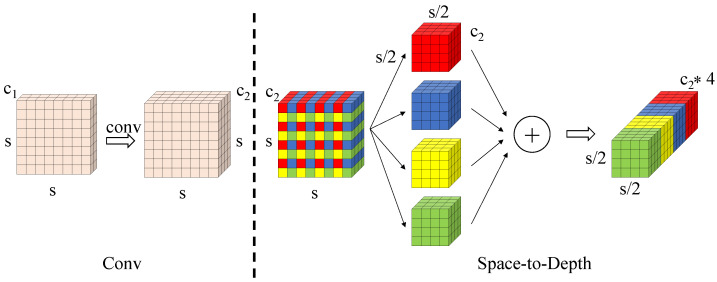
SPD-Conv convolutional structure diagram. The different colors are used solely for visual discrimination and do not represent any specific meaning.

This hierarchical transformation fundamentally addresses the limitations of traditional downsampling methods by achieving lossless conversion of spatial information into channel-wise representations. The resulting $s^{2}$-fold channel expansion effectively preserves the discrete spatial patterns of small targets, enabling deeper network layers to capture their geometric characteristics more reliably. Consequently, the SPD-Conv mechanism significantly mitigates feature degradation throughout the network architecture and substantially enhances small object detection performance, see Figure 2.

The parameters of SPD-Conv module used in this study are shown in Table 1.

#### 2.1.2. CBAM Attention Mechanism

**Figure 3:**
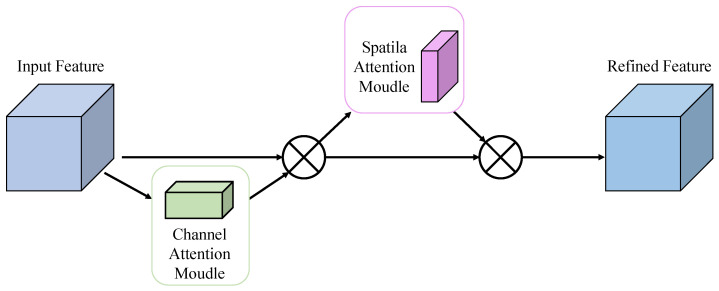
CBAM Attention Module Diagram.

Convolutional Block Attention Module (CBAM) is a lightweight feature enhancement module that integrates channel and spatial attention mechanisms in a dual-path structure, enabling adaptive feature refinement in convolutional neural networks. Its architecture, illustrated in Figure 3, employs a cascaded design to sequentially compute channel and spatial attention maps, thus enhancing the network’s performance and making model focus on important regions while keeping computational overhead minimal. The channel attention submodule exploits inter-channel relationships by employing both global average pooling and global max pooling, followed by shared multi-layer perceptrons (MLPs) to produce attention weights. This mechanism makes the model adaptively emphasize feature channels that are most linked to the given target. The spatial attention submodule utilizes 2D pooling operations along with a convolutional layer to generate spatial attention maps, which help direct the network’s focus toward semantically significant areas. In small object detection tasks, CBAM offers notable benefits: channel attention enhances the expression of weak signals, while spatial attention suppresses background noise, and their combined effect improves detection accuracy for small and subtle targets.

**Figure 4:**
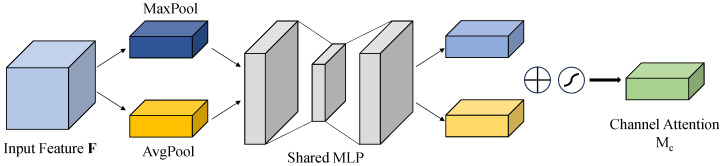
Channel Attention Module.

Details of the channel attention module are visually presented on Figure 4. This module applies global average pooling and global max pooling independently across the channel dimension, producing two complementary feature descriptors that capture both statistical characteristics and extreme responses. The extracted descriptors are subsequently processed through a shared two-layer fully connected network equipped with nonlinear activation functions, enabling the modeling of dependencies across channels. In the final step, those weights will be integrated into the origin feature map through using element-wise multiplication, thereby adaptively strengthening informative channels while reducing the impact of less relevant or noisy ones. This data-driven attention mechanism, leveraging both prior statistics and learned parameters, significantly improves the model’s channel-wise feature selection capability, especially in scenarios with weak or noisy signals.

**Figure 5:**
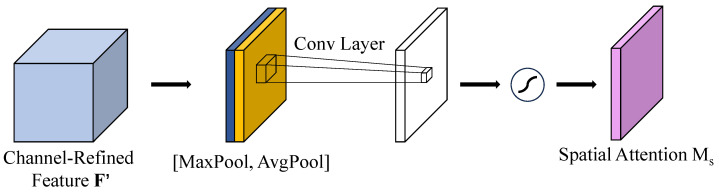
Spatial Attention Module.

The working principle of the spatial attention algorithm can be seen in Figure 5. This module employs a spatial feature aggregation strategy. The input feature maps are first processed through parallel channel-wise max pooling and average pooling operations, which extract extreme features with prominent spatial variations and statistical features, respectively, resulting in two complementary spatial response maps. The obtained descriptors are combined through concatenation and a spatial attention map is then derived by applying convolutional operations. Following normalization using the Sigmoid function, the obtained weights constrained within the range of [0, 1] are integrated into the input feature map through element-wise multiplication, thereby adaptively enhancing spatial characteristics. By combining both extreme and statistical spatial features, the approach refines the performance of the model to discern tiny defects against complex backgrounds, thus enhancing precision in identifying diminutive targets.

The parameters of CBAM module used in this study are shown in Table 2.

#### 2.1.3. BiFPN Feature Extraction Network

**Figure 6:**
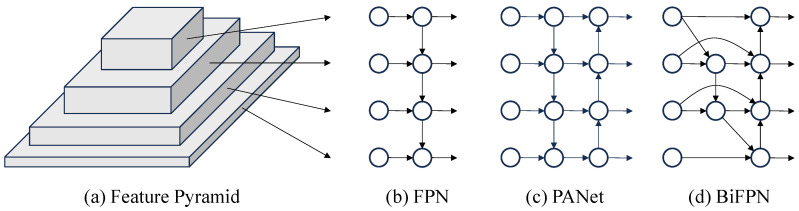
Structure of Feature Extraction Network.

As a critical step in object recognition, feature extraction determines the performance of a model to extract discriminative patterns, where the design of the extraction mechanism plays a key role in enhancing both perceptual accuracy and robustness across diverse scenarios. As research has progressed, the paradigm has evolved from single-path deep abstraction to multi-scale feature fusion, as displayed in Figure 6. Traditional convolutional neural networks adopt a bottom–up architecture, where high-level semantic features are progressively extracted through stacked convolution and pooling layers, as shown in Figure 6a. However, this unidirectional propagation introduces a significant semantic gap: as the network deepens, high-resolution low-level features lose spatial detail due to repeated downsampling, while deep low-resolution features, despite their rich semantics, lack the geometric detail necessary for precise object localization. This semantic–spatial gap degrades small-object detection.

To address this limitation, scholars have introduced network structures like the Feature Pyramid Network (FPN) and Path Aggregation Network (PANet), which are depicted in Figure 6b,c, respectively. These architectures incorporate top–down pathways along with lateral connections to facilitate multi-scale feature fusion, thereby efficiently narrowing the semantic disparity between deeper and shallower network layers. FPN improves upon conventional feature extraction by preserving the bottom-up backbone and introducing a top–down pathway that integrates low-layer spatial details with high-layer semantic message via lateral connections. It allows the network to maintain rich semantic understanding while achieving accurate spatial localization. Building upon FPN, PANet introduces a bottom-up path to form a bidirectional enhancement strategy. By feeding low-level geometric details back into higher layers, PANet constructs a closed-loop optimization mechanism that improves semantic consistency in shallow features and enhances the localization accuracy of deeper layers. This dual-path design achieves a dynamic balance between semantic abstraction and spatial precision.

Building upon PANet, BiFPN further enhances feature fusion efficiency through architectural refinement and parameter optimization. First, it employs a node reduction strategy to eliminate redundant connections with only a single input or output, forming a more lightweight and streamlined topology. Then, cross-scale skip connections are introduced to establish bidirectional information flow between nodes at the same resolution, improving feature reuse. Finally, a learnable weighted fusion mechanism assigns adaptive weights to features at different scales, enabling dynamic calibration across levels. This dual optimization in structure and parameters reduces computational cost. In addition, it enhances semantic consistency across scales, delivering superior ability in dense small object detection tasks. The structure is illustrated in Figure 6d.

The parameters of BiFPN module used in this study are shown in Table 3.

#### 2.1.4. Detection Head for Small Defects

The biggest difficulty in distribution line recognition is that the defect coverage in the original image is significantly limited, which can lead to serious feature loss. YOLOv11n uses three detection heads at 80×80, 40×40, and 20×20 (for a 640×640 input). Original image information is extracted into the three detection heads through feature extraction network, and smaller detection head sizes correspond to deeper semantic information. Finally, the model will integrate the information from the three detection heads and obtain the final recognition result. During the extraction process, the loss of features will cause the model to lose information about small target defects.

**Figure 7:**
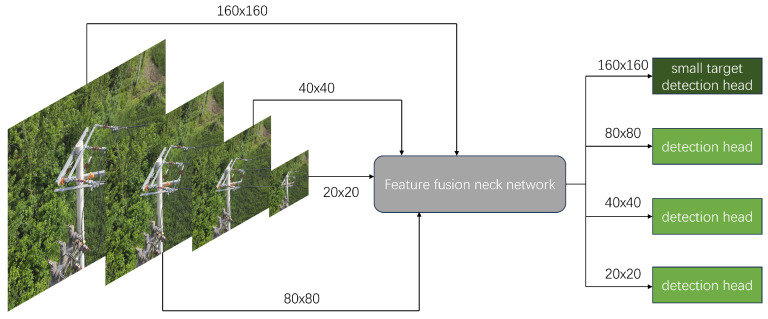
Small Target Detection Head.

Therefore, for small target defects, we add a 160×160 high-resolution inspection head and the corresponding hierarchical structure, which is displayed in Figure 7. The TDD-YOLO fuses the shallow detail information of the fourth layer of the backbone with the upsampled deep semantic information of the last layer in Neck through BiFPN to obtain the detection head dedicated to small target defects. This structure fully combines the richness of shallow detail features and the abstraction of deep semantic information, which marginally increases the model’s complexity but greatly enhances the model’s recognition performance for tiny target defects.

### 2.2. Fully Autonomous Defect Detection System

#### 2.2.1. Drones and Embedded Platform

**Figure 8:**
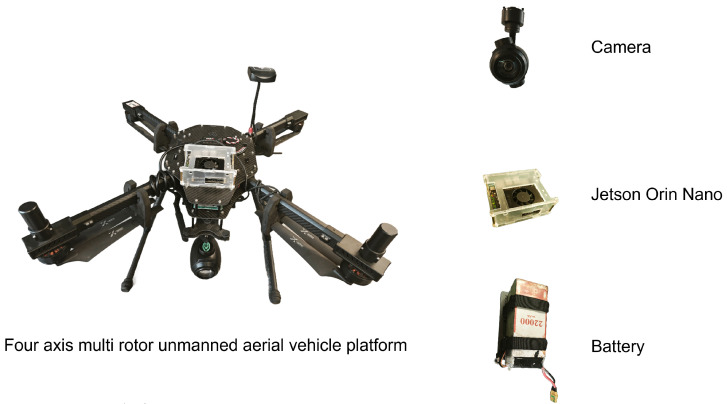
UAV Platform.

As shown in Figure 8, this study conducted flight experiments using a quadrotor experimental platform independently developed in the laboratory in order to validate the performance of the proposed improved model in detecting defects within real-world application scenarios. The platform is equipped with a six-axis inertial sensor, magnetometer, GPS module, data logging module, aircraft status indicator lights, and a battery unit. Additionally, it integrates an NVIDIA Jetson Orin Nano (4 GB RAM) edge computing unit (up to 20 TOPS) to support the deployment of the proposed model for defect detection.

We chose NVIDIA Jetson Orin Nano as the airborne edge computing device because of its small size, low power consumption and strong CUDA support. Its 20 Tera Operations Per Second (TOPS) computing power can detect towers in real-time based on a 25 Frames Per Second (FPS) video stream, supporting real-time, reliable operation.

The specific information of the equipment is shown in the Table 4.

#### 2.2.2. System Framework

**Figure 9:**
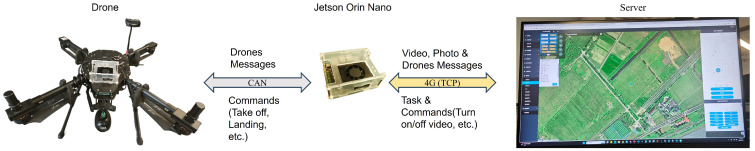
System framework.

The fully autonomous tower defect detection system adopts the following communication framework. The drone communicates with the Jetson Orin Nano (NVIDIA Corporation, headquartered in Santa Clara, CA, USA) via CAN protocol, and the Jetson Orin Nano communicates with the server through its loaded 4G module. Among them, drone information, tasks, and instructions are transmitted through TCP protocol to ensure the reliability of important data transmission. The video stream is transmitted to the server through UDP protocol to ensure the real-time performance of the system. This system communication structure can maximize the reliability and real-time performance of fully autonomous tower defect detection, see Figure 9.

We have developed a Graphical User Interface (GUI) for the server, which aims to display the system status more specifically, making it easier for non professionals to operate and maintain. The main functions are as follows:

1. Real-time display of status information from drones, video streams from cameras, and recognition results of fully autonomous tower defect detection.
2. Integrated graphical operation commands (such as turning on/off video streams, take-off, landing, remote control of drones, etc.).
3. Save the videos and photos taken during the flight for future analysis.

The autonomous system utilizes a 4G LTE link for command and control, as well as for transmitting critical data, such as detected defects, telemetry to the ground station. To address the latency and reliability requirements of real-time operation, the following measures were implemented:

- Telemetry and control signals are given the highest priority. The transmission of all processed images is initiated only once the connection is sufficiently stable.
- A continuous heartbeat signal is used to monitor link integrity. Upon a timeout, the system triggers a pre-programmed fail-safe maneuver, which can be set to automatic return-to-home or hover.
- The performance of the 4G link was rigorously evaluated during field tests. Key metrics, including end-to-end latency, packet loss rate, and average uplink/downlink bandwidth, were logged for analysis. These quantitative results are presented in Section 3.5 to validate the suitability of the communication system for the intended application.

The model’s actual end-to-end processing latency is essential for the fully autonomous defect inspection system. In our practical deployment on the Jetson Orin Nano platform, we reduced this latency by converting the model into an ENGINE format with FP16 precision.

The autonomous inspection system operates through a structured four-stage process. After initialization and vertical ascent for tower height calculation, the drone navigates to each tower, hovers directly above to capture images of the tower head, and executes our defect detection model in real-time. The system cyclically proceeds to subsequent towers along the power line direction until all specified towers are inspected, and then autonomously returns. Manual operator intervention is enabled to handle unexpected situations, ensuring operational robustness.

#### 2.2.3. Autonomous Flight Process Details

The visual input for tower detection comprises a 1080p resolution video stream at 25 FPS. For this purpose, we utilize the YOLOv8n model, which is separated from the core defect detection model introduced in this paper. Given that our research emphasizes the advancement of defect detection methodologies, the tower detection model, being a prerequisite component, falls outside the scope of detailed discussion in this study. The specific task process is as follows:

1. Preparation stage: This stage initializes the drone, the onboard Jetson Orin Nano computer, and the communication link with the ground station server. Upon successful initialization, the system interface displays real-time drone status and the camera video feed. The operator can then adjust the drone’s position and, by visually confirming that the target tower route is within the video stream, manually activate the tower recognition module. Once these preparatory checks are satisfactory, the operator issues the command to commence the autonomous inspection task.
2. Take-off phase: At the mission outset, the drone ascends vertically while continuously monitoring the tower in real time. It performs two hovering maneuvers at positions where the complete tower is within the recognition frame and the tower head is centered. The tower’s height is calculated through photogrammetric analysis of these two images in conjunction with camera parameters. Subsequently, the drone ascends an additional 10 m beyond the calculated height to attain a safe operational altitude.
3. Detection stage: The drone navigates toward the target tower, dynamically adjusting its camera gimbal based on real-time tower detection feedback to maintain continuous tracking. Upon reaching a position directly above the tower, the drone enters a stable hover to capture high-resolution images of the tower head. These images are then automatically processed by the defect detection model proposed in this work to identify and localize critical defects.
4. Cycle stage: After completing the target tower detection, the system will search for the next tower along the line based on the cable direction and repeat the actions of the detection stage.
5. Tower detection: The tower inspection process comprises three types: the tower head, the tower body, and the entire tower. During the preparation and take-off phases, the tower body and the entire tower are detected to calculate a safe flight distance and tower orientation. The inspection of the tower head, in turn, provides the basis for determining whether the drone is correctly positioned above the tower. Finally, during the cyclic inspection phase, the drone utilizes the inspection data of the entire tower to determine the azimuth for proceeding to the next one.
6. Exception handling: Exception handling is categorized into two types. The first type involves system-level issues, including communication links, the AI model, or the UAV’s own status. When these occur, the UAV initiates a predefined contingency procedure, typically opting to hover or perform an automated return-to-home. The second type comprises logic anomalies, such as detecting a corner tower, finding no tower in the video feed, or identifying multiple towers, where the system lacks the autonomy to proceed. These anomalies trigger an alert in the GUI, requiring operator assistance and human-in-the-loop decision-making to resume the task.

The task parameters specify the quantity and identification numbers of the towers to be detected. Once the defect inspection of all towers is finished, the UAV autonomously returns to the take-off point. This hovering action, which involves capturing photos above each tower, serves to document the actual geographic coordinates and associate them with the respective tower ID, thereby streamlining future maintenance operations.

### 2.3. Dataset Description

Because of the absence of related public datasets, we established a customized dataset comprising inspection flight imagery supplied by a power grid corporation, supplemented with images gathered from online repositories. This dataset encompasses two critical defect types: missing tension clamp shells and improperly tied insulators. The dataset was labeled through LabelImg. A total of 6869 images were collected and split 8:1:1 into train/val/test. Specifically, the training set contains 5495 images, while both the validation and test sets consist of 687 images each, as summarized in Table 5. Among the annotations, there are 11,472 instances of improperly tied defects and 13,039 instances of missing protective shells, covering a wide range of defect types under various backgrounds and UAV perspectives. This comprehensive dataset ensures the adaptability of the model in real-world power distribution line inspection scenarios.

The defect distribution in the dataset is a direct consequence of the UAV inspection scenario. To maintain a safe distance from the utility tower, the UAV captures images where defects invariably appear as small targets. As shown in Table 6, the majority of defects (66.86%) fall within the 0.1–1% object-to-image area ratio range, comprising 16,387 target instances. An additional 31.02% of defects are even smaller, with area ratios below 0.1%, accounting for 7603 targets. Notably, medium-sized defects (1–10% area ratio) represent only 2.13% of the dataset (521 targets), while no large defects (10–100% area ratio) are present. This distribution highlights the dataset’s predominant focus on the challenging problem of small object detection.

**Figure 10:**
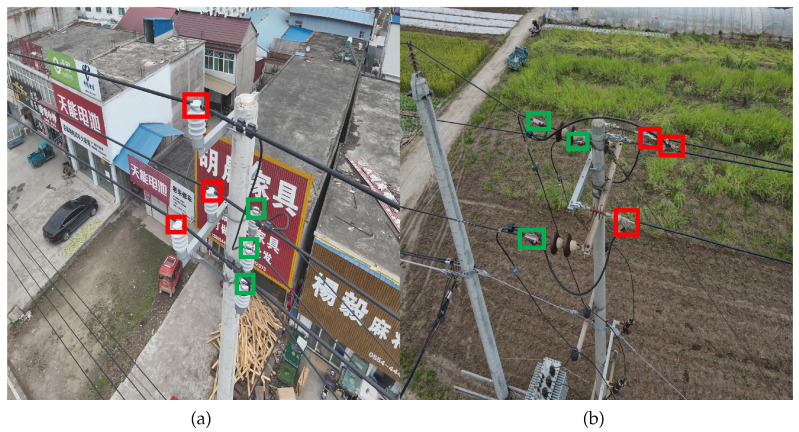
Schematic diagram of target defects in power distribution. (**a**) Non-standard insulator binding and (**b**) missing tension clamp shell. The red boxes in the picture indicate the defective parts, and the green boxes indicate the normal parts.

To visually illustrate the two defect types, representative examples are shown in Figure 10. In Figure 10a, red boxes indicate ‘non-standard binding’ defects, while green boxes denote ‘standardized binding’. Normally, distribution cables should be placed and bundled in the grooves of the insulator to ensure that the insulator can support the distribution cable and provide electrical insulation. When the lashing is not standardized, the insulator cannot provide support and electrical protection for the cable, and it cannot guarantee reliable operation of the distribution line. In Figure 10b, red boxes highlight ‘missing tension clamp protective shells’, and green boxes correspond to normal ones. Tension clamps are critical for securing power distribution cables. The absence of a protective cover leaves the tension clamp completely exposed, making it vulnerable to environmental corrosion. Once corrosion or aging occurs, there is a risk of the distribution cable will fall off. The protective cover of the tension clamp provides isolation and protection, effectively extending the service life of the tension clamp.

To further explore the effectiveness and advantages of TDD-YOLO, comprehensive experiments were carried out across a range of challenging scenarios to assess its adaptability and performance stability. The defect detection test set was processed using four representative image enhancement approaches:

1. motion blur, to simulate dynamic scenarios caused by UAV movement.
2. increased brightness, to mimic direct sunlight exposure.
3. increased brightness, representing overcast conditions.
4. haze addition, to emulate foggy weather conditions.

These environmental perturbations enable systematic evaluation of TDD-YOLO’s performance to practical distribution line inspection tasks. Details of image enhancement approaches are displayed in Table 7.

### 2.4. Training Details

Model training was conducted with an Intel Core i5-13400 processor and an NVIDIA RTX 4070Ti GPU. Parameter settings and training environment are displayed in Table 8 and Table 9. According to results of pre-training, the model we introduced began to converge around epoch 200. Therefore, we set epoch to 300. Due to hardware constraints, the batch size was limited to 16.

The detailed training parameters employed in our experiments are presented in Table 9. The model underwent comprehensive training for 300 epochs, with a consistent batch size of 16. We adopted the Adam-W optimizer, known for its effective handling of sparse gradients and improved generalization capability, with an initial learning rate set to 0.001. All input images were uniformly resized to 640×640 pixels to maintain consistency in feature extraction. This carefully optimized configuration ensures fair comparison across different model variants in our ablation study while maintaining training stability and convergence efficiency.

### 2.5. Evaluation Metrics

Standardized evaluation metrics are critical for the objective assessment of recognition algorithms, and we employ them to evaluate the performance of the model, including mean average precision (mAP), precision (*P*), recall (*R*), parameters, frames per second (FPS), giga floating-point operations per second(GFLOPs) and model size.

Among the evaluation metrics, mAP is regarded as the primary indicator, providing a balanced assessment of precision and recall across multiple object categories. It is computed by first determining the average precision (AP) for each individual class, followed by calculating the average of all class-specific AP values, as defined in Equation (1). This hierarchical averaging approach ensures fairness across categories and offers a holistic view of the model’s detection ability in complex scenarios.

(1) $$mAP=\frac{1}{N}\sum_{i=1}^{N}AP_{i}$$

There are two common evaluation metrics for mAP: mAP@0.5 and mAP@0.5:0.95. mAP@0.5 is calculated using a fixed IoU threshold of 0.5, meaning a predicted bounding box is classified as correct when its overlap with the corresponding ground truth box meets or exceeds this threshold. For each category, the average precision is determined separately, and the final metric is derived by averaging these results across all classes. On the other hand, mAP@0.5:0.95 employs a dynamic IoU threshold ranging from 50% to 95%, with a 5% step. The average precision is calculated at each threshold and then averaged across the range.

In the computation process, the AP for each class is derived from the precision-recall curve, where recall is plotted on the x-axis and precision on the y-axis. The mathematical formulation for this calculation is presented in Equation (2).

(2) $$AP=\int_{0}^{1}P\left(R\right)dR$$

Precision is calculated as the fraction of recognized positive instances among predictions labeled as positive. It highlights the effectiveness of model in minimizing false positives. Conversely, recall is expressed as the fraction of identified positive instances out of actual positives, showing the capability of model to avoid missed detections. This metric reflects the model’s capability to differentiate actual positives from incorrectly pretdect as negatives, with a focus on reducing errors where negative samples are mistakenly identified as positive. The mathematical expressions for *R* and *P* are presented in Equations (3) and (4), respectively.

(3) $$Recall=\frac{TP}{TP+FN}$$

(4) $$Precision=\frac{TP}{TP+FP}$$

In these computations, True Positive (TP) denotes cases where positive instances are correctly recognized by the model, False Positive (FP) indicates that negative instances are incorrectly recognized as positive, and a False Negative (FN) occurs when a positive instance is mistakenly classified as negative. From the confusion matrix, the evaluation metrics are intuitively obtained. It is displayed in Table 10.

We measure computational complexity in Giga Floating Point Operations (GFLOPs) for a single forward pass with batch a size of 1 and input resolution of 640×640.

This study establishes a multidimensional model evaluation system by integrating mean Average Precision (mAP), Parameters, GFLOPs and Size. The mAP reflects detection accuracy, Parameters indicates the total number of learnable parameters, and Size measures the overall model size. This analysis of the trade-off between precision and efficiency offers a robust basis for assessing the model’s effectiveness in this study.

To ensure statistical reliability, we performed five independent experiments with different random seeds for each model configuration. The precision, recall, and mAP metrics reported in this paper are the averages across these 5 runs. All values are presented with three decimal places.

## 3. Results

### 3.1. Ablation Experiment

To evaluate the effectiveness of proposed enhancement methods in defect detection, this study adopts YOLOv11n as the baseline model and conducts ablation experiments by comparing it with each improvement strategy. This comparison helps validate whether the modifications lead to improvements in detective metrics, which are vital for assessing object detection performance. Table 11 presents the experimental results, with checkmarks indicating the application of specific enhancements to the original YOLOv11n architecture.

Firstly, the convolution module was replaced with SPD Conv, increasing mAP@0.5 to 0.842 and mAP@0.5:0.95 to 0.481. This indicates that SPD Conv effectively preserves local features by converting spatial information into depth information. Secondly, replacing the Convolutional block with the Parallel Spatial Attention (C2PSA) module with the CBAM attention module increased mAP@0.5 to 0.841 and mAP@0.5:0.95 to 0.469. Meanwhile, both the model size and computational complexity decreased to a certain extent. This indicates that for small target defects, CBAM’s channel and spatial dual attention are more effective in directly enhancing local details compared to C2PSA’s multi-scale grouped convolution, which is particularly important for low-resolution small targets.

It is worth noting that the original framework of YOLOv11n was first subjected to SPPF processing before C2PSA. When replacing C2PSA with CBAM, we performed CBAM first and then SPPF, but achieved better results. The results of the two models are displayed in Table 12.

After modifying the module order, replacing C2PSA with CBAM increased the mAP@0.5 by 0.6% percentage points. The reason is that SPPF blurs local details through multi-scale pooling, which directly affects the feature extraction of small targets. If CBAM is later connected, spatial attention may fail due to reduced feature map resolution. CBAM can prioritize retaining details of key areas in the front. Therefore, in the end, while replacing the C2PSA module, we chose to adjust the model framework, resulting in better recognition performance.

Finally, the feature extraction network was reconstructed by using BiFPN, and a detection head with a size of 160×160 was integrated, achieving mAP@0.5 = 0.856 and mAP@0.5:0.95 = 0.489. BiFPN connects high-level semantic information and low-level spatial information through bidirectional fusion, thereby enhancing low-level semantic information and high-level spatial information, and improving the performance of the model in identifying small target defects. The added 160 × 160 detection head preserves more local details for the model, which is crucial for detecting small objects.

Ablation experiments showed that all the improved modules we proposed made a positive contribution to performance, with mAP@0.5 increased to 0.873 and mAP@0.5:0.95 increased to 0.508.

### 3.2. Comparison Experiment

This study assesses the effectiveness of TDD-YOLO by benchmarking it against existing models. The proposed TDD-YOLO was evaluated against several state-of-the-art lightweight object detection models, including YOLOv7-tiny, YOLOv8n, YOLOv9-tiny, YOLOv10n, and YOLOv11n, under standardized experimental conditions. As summarized in Table 13, TDD-YOLO significantly outperformed all other lightweight YOLO variants across multiple core detection metrics. Specifically, it achieved an mAP@0.5 = 0.872, an mAP@0.5:0.95 = 0.50.8, and ran at 29 FPS on the Jetson Orin Nano.

While YOLOv9-tiny and YOLOv10n exhibited competitive detection accuracy, their inference speeds were considerably lower than that of TDD-YOLO on the same hardware. Notably, YOLOv9-tiny, despite having the closest recognition performance to our model, attained only 12 FPS. System profiling revealed that YOLOv9-tiny failed to fully utilize the available GPU resources, operating at approximately 60% utilization, which indicates potential architectural or software-level incompatibilities with the Jetson platform. Other models with faster frame rates, such as YOLOv8n, exhibited significantly inferior detection accuracy compared to TDD-YOLO.

Overall, TDD-YOLO achieved the most effective balance between recognition performance and inference speed among the evaluated models, thereby enhancing the reliability of the fully autonomous, real-time distribution tower defect detection system.

Considering that the stronger the model recognition performance, the more effective objects are recognized, and that introducing post-processing is difficult to accurately express the model recognition speed, FPS only calculates the preprocessing and inference parts.

### 3.3. Robustness Experiment

**Figure 11:**
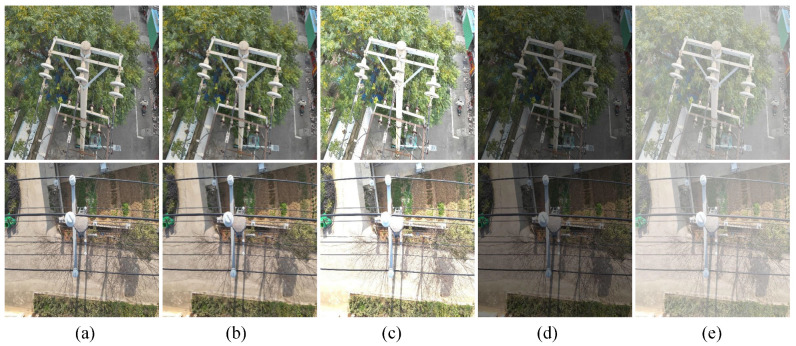
Image Comparison under Different Image Enhancement Operations. (**a**) Original. (**b**) Motion Blur. (**c**) Increased Lightness. (**d**) Decreased Lightness. (**e**) Haze Addition.

To rigorously assess model robustness, we subjected both the baseline and the proposed TDD-YOLO to the simulated degraded conditions outlined in Section 2.3, including motion blur, extreme brightness, and haze. The results, compared in Figure 11, reveal that TDD-YOLO achieves consistently higher detection accuracy. This demonstrates its superior capability in mitigating the effects of key photometric variations and atmospheric disturbances, a crucial advancement for dependable UAV inspection systems.

In this experiment, the val set which has been processed was used, and the test results are shown in Table 14. Experimental results show that TDD-YOLO is significantly better than the benchmark model (YOLOv11n) in the original image and a variety of visual degradation scenarios. When no perturbation is added, the mAP@0.5 of TDD-YOLO reaches 87.3%, which is 3.9% higher than that of the benchmark model, and the mAP@0.5:0.95 increased by 4.1%. In the motion blur, abnormal brightness and fogging interference scenarios, the mAP@0.5 index remained 83.2%, 82.9, 84.7%, and 74.4%, respectively, which was 3.5–4.7% percentage points higher than that of the benchmark model. These results indicate stronger environmental robustness.

The experiment verified the reliability of TDD-YOLO in complex distribution tower detection scenarios, providing a more reliable guarantee for unmanned aerial vehicle power distribution detection.

### 3.4. Portability Experiment

The improvement methods we proposed were applied on the YOLOv8n model to verify its advantages and universality in enhancing the performance of detecting small objects because YOLOv8n is currently the most representative version of YOLO and also one of the most widely used YOLO versions on edge devices. The results of the portability experiment are displayed in Table 15.

The benchmark model YOLOV8n achieved mAP@0.5 of 0.687 and mAP@0.5:0.95 = 0.343. After adding the CBAM attention module, the performance was slightly improved, reaching mAP@0.5 of 0.698 and mAP@0.5:0.95 = 0.343. After replacing the convolution module with SPD Conv, the model performance was significantly improved, reaching mAP@0.5 = 0.748 and mAP@0.5:0.95 = 0.384, but the number of parameters also increased the most. After modifying the feature extraction network to BiFPN and adding a detection head, the performance improvement was also significant, reaching mAP@0.5 of 0.733 and mAP@0.5:0.95 = 0.382, with a slight increase in size, balancing model complexity and performance. After applying all improvement methods simultaneously to YOLOv8n, mAP@0.5 increased by 8.8% and mAP@0.5:0.95 increased by 7.2%. The number of parameters increased by 0.8 M; a modest increase in parameters (+0.8 M) yielded substantial gains, achieving a balance between detection performance and efficiency.

The experimental results show that the proposed method has universality and can enhance the detection accuracy of small target objects under different YOLO frameworks.

### 3.5. Flight Experiment

**Figure 12:**
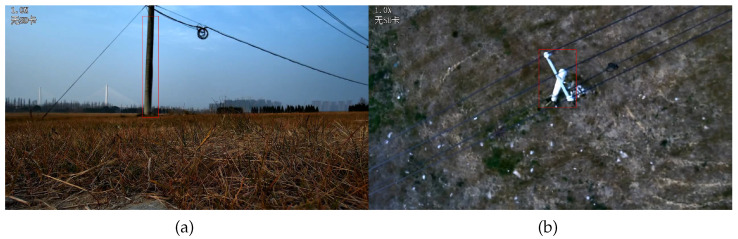
Tower detection. (**a**) Real-time identification of the tower body and (**b**) the tower head, with red bounding boxes indicating the detected regions.

**Figure 13:**
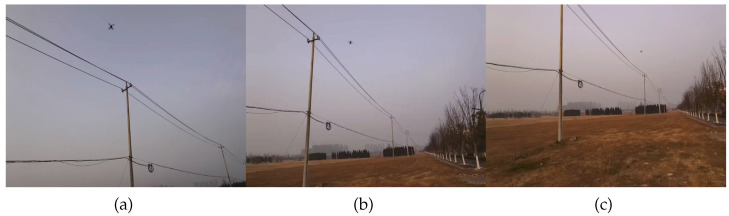
Autonomous flight process. (**a**) UAV inspecting the current transmission tower, (**b**) autonomously navigating to the next tower, and (**c**) inspecting the subsequent tower.

**Figure 14:**
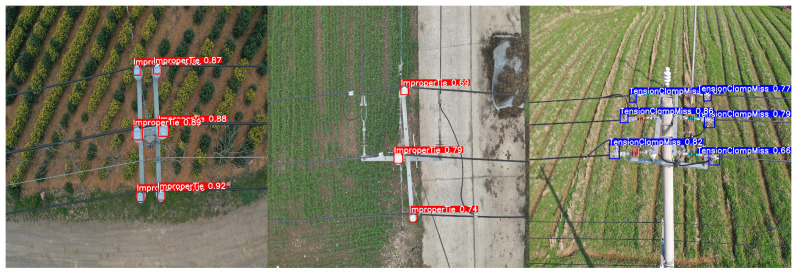
Flight experiment result.

The system proposed in this article was used for defect detection of tower lines, and the results are as below: Figure 12 (Tower body detection) shows that in ground preparation mode, the aircraft identifies the tower based on the video stream. Once the tower is identified and the operator sends command to start detect task, the drone will start operating autonomously. Figure 12 and Figure 13a (tower top detection) show the drone flying towards and detecting the target tower. Figure 13b,c show the drone flying towards the target tower to be inspected at the next level and conducting defect detection on the tower at the next level. Figure 14 shows the defect detection results in field runs.

The defect detection results for small targets in real-world applications using the proposed model are shown in the Figure 14.

As outlined in Section 2.2.2, the reliability of the 4G link is critical for safe operation. The link performance was evaluated over 5 separate flight missions in a typical operational environment. Furthermore, the actual operational speed of the TDD-YOLO model was benchmarked under in-flight conditions. For deployment, the model was converted to the ENGINE format and utilized FP16 precision to enhance computational efficiency. The results are summarized in Table 16.

The data confirm that the 4G link consistently met the predefined thresholds for real-time control and data transmission. The observed latency and packet loss are sufficiently low to ensure stable drone command and timely reception of defect alerts. The occasional fluctuations in bandwidth did not impact the mission, as the data prioritization scheme ensured that critical messages were always transmitted.

The flight experiment results show that the proposed system and model perform well in practical scenarios. It can accurately detect two types of defects in autonomous flight operations improper insulator binding and missing tension clamps, improving the safety of traditional power tower line inspection processes and saving a lot of manpower resources for tower defect detection. Flight trials show accurate detection of the two target defects during autonomous operation.

## 4. Discussions

### 4.1. FP/FN Analysis and Future Directions

The majority of false positives occur when complex background structures are mistaken for defects. For instance, shadows cast by cross-arms or textured patterns on rusted tower metal were occasionally misclassified as “non-standard binding”. The most challenging cases for our model are defects with extremely low pixel area and contrast. Heavy occlusion is another significant factor leading to false negatives. When defects are partially hidden by other wires or components, the model often fails to recognize them.

Based on this error analysis, we identify several promising directions for future work:

- To mitigate false positives, future datasets could be augmented with a wider variety of background challenges (e.g., more shadow patterns, complex textures) and hard negatives (non-defective components that are visually similar to defects).
- Advanced Attention Mechanisms: To reduce false negatives, especially for low-contrast defects, exploring more powerful attention mechanisms that can better focus on subtle, local discrepancies within a cluttered scene could be beneficial.
- For the smallest defects, experimenting with higher-resolution input images or adaptive patch-based detection could provide the necessary pixel-level details to improve recall, albeit at a potential computational cost.

### 4.2. Device Support

In the portability experiment, we validated the proposed method on YOLOv8n and demonstrated its generality across different architectures. This provides an important basis for selecting different models on different devices. Combined with the results of comparative experiments, if other edge computing devices with lower computational power are selected, we recommend using the improved model of YOLOv8n as the defect detection model. Subsequent work should also explore the deployment effect on other edge computing devices.

### 4.3. Limitations in Environmental Robustness and Future Data Enhancement

Considering that different weather conditions may be encountered in actual operations, we process the dataset with motion blur, increased brightness, reduced brightness and haze addition methods to validate the robustness of the proposed model. The greatest impact on model accuracy is observed with the introduction of haze. Atomization processing leads to the enhancement of low-frequency components and attenuation of high-frequency details in the image, resulting in the partial or complete disappearance of key features that the model relies on (such as texture, edges, shape), which is not conducive to the recognition of small target defects by the model. Although the model performs well compared to the original model in this situation, the detection accuracy is not ideal due to the limitations of the dataset, only including data from normal weather. In addition, most of the target types are small target defect types, and there are few large-scale detection targets. Therefore, in future work, it is necessary to enrich the weather types of the dataset and increase the proportion of large-scale targets to enhance the multi-scale detection ability and robustness of the model.

### 4.4. Addressing Class Imbalance

We identified a notable performance disparity between the “improper tie” and “missing clamp” defect classes, with the mAP for the latter being approximately 10% lower in our initial experiments. We attribute this performance gap primarily to the inherently smaller physical size of the “missing clamp” defects, which makes them more susceptible to feature loss during network down-sampling and thus more challenging to detect. To mitigate this bias, we strategically increased the weighting factor of the classification loss. This adjustment compels the model to focus greater attention on these challenging, small-size examples during training. The experimental results confirm the effectiveness of this approach: the performance gap between the two defect classes was significantly narrowed, while the overall mAP also received a concomitant boost.

The results, as comparatively detailed in Table 17, demonstrate the efficacy of this strategy. Not only did the revised training objective lead to a more balanced performance improvement between the two defect classes, effectively narrowing the performance gap, but it also contributed to a further increase in the overall mean Average Precision (mAP). This outcome confirms that mitigating class imbalance is crucial for enhancing the robustness and fairness of our defect detection system. Future work will delve deeper into this direction, exploring more sophisticated strategies such as Focal Loss techniques to achieve even greater class-balanced performance.

## 5. Conclusions

This study presents and validates a complete, edge-deployed system for the fully autonomous and real-time inspection of distribution towers, marking a significant step toward the intelligent and automated maintenance of power grid infrastructure. The core of this system, the TDD-YOLO model, successfully bridges the critical gap between high-accuracy defect detection and practical deployment on resource-constrained platforms, as demonstrated by its robust performance on the NVIDIA Jetson Orin Nano.

The primary significance of this work is three-fold. First, from a methodological perspective, it moves beyond isolated model improvements by offering a principled, synergistic framework for small-target defect detection. The integrated pipeline of SPD-Conv, CBAM, BiFPN, and a high-resolution head provides a reusable blueprint for tackling the compounded challenges of information loss, low contrast, and inefficient feature fusion in other industrial inspection domains. Second, in terms of practical application, this research delivers a tangible solution that transitions defect inspection from a manual, labor-intensive process to a seamless autonomous operation. This has the direct potential to enhance grid safety, reduce operational costs, and prevent catastrophic failures by enabling proactive and frequent inspections. Third, its engineering value is proven through a real-world, edge-computing implementation, confirming that complex deep learning models can be effectively leveraged for real-time analysis in challenging field environments.

While the current system demonstrates considerable promise, future work will focus on three key areas to broaden its impact: (1) enhancing operational efficiency through model compression techniques like pruning to democratize its use across an even wider array of edge devices; (2) architecturally integrating the separate tower recognition and defect detection models into a single, streamlined network to simplify the deployment pipeline; and (3) expanding the diversity and scale of the defect dataset to improve the model’s generalizability and robustness across various geographical and environmental conditions.

## Figures and Tables

**Table 1:** SPD-Conv details.

Parameters	Value
Scale factor	2
Conv kernel Size	3×3
Conv stride	1
Conv padding	1

**Table 2:** CBAM Details.

Parameters	Value
Reduction	16
Kernel size	7×7

**Table 3:** BiFPN Details.

Parameters	Value
Number of repeats	1
Number of input	2 or 3
Weighted fusion	True

**Table 4:** Platform Details.

Name	Parameter
Drone	Self developed quadcopter unmanned aerial vehicle
Computing unit	an NVIDIA Jetson Orin Nano (4 GB RAM, 20 TOPS)
Camera	1080p Camera, 25 FPS

**Table 5:** Dataset details.

Name	Counts
Total picture	6869
train	5495
val	687
test	687
ImproperTie instance	11,472
TensionClampMiss instance	13,039

**Table 6:** Defect distribution details.

Object-to-Image Area Ratio (%)	Target Counts	Proportion (%)
0–0.1	7603	31.02
0.1–1	16,387	66.86
1–10	521	2.13
10–100	0	0

**Table 7:** Details of image enhancement approaches.

Name	Parameter	Value
Motion blur	Kernel size	15
	Angle range (degrees)	0–120
	Direction	−1.0–1.0
Increased/Decreased brightness	Brightness scale factor	0.5–1.5
Haze addition	Haze factor	0.01–0.3
	Lightness constant	0.8–1.2

**Table 8:** Training environment.

Name	Parameter
CPU	13th Gen Intel(R) Core(TM) i5-13400
GPU	NVIDIA GeForce RTX4070Ti
Memory	32 GB

**Table 9:** Training parameters.

Parameter	Value
epoch	300
batch-size	16
optimizer	AdamW
learning-rate	0.001
image-size	640×640

**Table 10:** Confusion matrix.

Predicted/Ground Truth	Positive	Negative
Positive	TP	FP
Negative	FN	TN

**Table 11:** Results of ablation experiment.

Model	SPD-Conv	CBAM	BiFPN	Detector Head	mAP@0.5	mAP@0.5:0.95	Size (M)	GFLOPs
YOLOv11n					0.835	0.466	5.5	6.3
A	✔				0.842	0.480	6.0	12.6
B		✔			0.841	0.469	5.1	6.2
C			✔	✔	0.856	0.489	6.4	11.3
D	✔	✔			0.860	0.492	5.6	12.4
E		✔	✔	✔	0.859	0.491	6.0	11.2
F	✔		✔	✔	0.870	0.505	6.0	18.4
TDD-YOLO	✔	✔	✔	✔	0.873	0.508	6.1	18.5

✔ indicates the corresponding module is added.

**Table 12:** Comparison of models.

Model	Precision	Recall	mAP@0.5	mAP@0.5:0.95	Size (M)	GFLOPs
Origin frame	0.879	0.747	0.835	0.465	5.1	6.2
Modified frame	0.878	0.762	0.841	0.471	5.1	6.2

**Table 13:** Results of comparison experiment.

Model	Precision	Recall	mAP@0.5	mAP@0.5:0.95	Size (M)	GFLOPs	FPS
EfficientDet-D0	0.732	0.588	0.640	0.282	15.1	4.0	26
YOLOv7-tiny	0.773	0.609	0.675	0.319	12.3	13.7	37
YOLOv8n	0.760	0.631	0.687	0.343	6.3	8.2	40
YOLOv9-tiny	0.905	0.785	0.869	0.505	6.1	11.0	12
YOLOv10n	0.893	0.785	0.865	0.496	5.8	8.2	28
YOLOv11n	0.876	0.749	0.835	0.466	5.5	6.3	31
YOLOv12n	0.817	0.692	0.749	0.378	5.5	5.8	36
TDD-YOLO	0.886	0.801	0.873	0.508	6.1	18.5	29

**Table 14:** Recognition performance under different image enhancement operations.

Image Operation	Model	Precision	Recall	mAP@0.5	mAP@0.5:0.95
Original Image	YOLOv11n	0.876	0.749	0.834	0.467
Original Image	TDD-YOLO	0.886	0.801	0.873	0.508
Motion blur	YOLOv11n	0.838	0.725	0.797	0.449
Motion blur	TDD-YOLO	0.877	0.753	0.832	0.481
Increased brightness	YOLOv11n	0.836	0.706	0.782	0.421
Increased brightness	TDD-YOLO	0.878	0.751	0.829	0.469
Decreased brightness	YOLOv11n	0.872	0.717	0.803	0.442
Decreased brightness	TDD-YOLO	0.871	0.771	0.847	0.485
Haze addition	YOLOv11n	0.816	0.612	0.699	0.372
Haze addition	TDD-YOLO	0.817	0.672	0.744	0.418

**Table 15:** Results of portability experiment.

Model	SPD-Conv	CBAM	BiFPN	Detector Head	mAP@0.5	mAP@0.5:0.95	Size (M)	GFLOPs
YOLOv8n					0.687	0.343	6.3	8.2
A	✔				0.748	0.384	6.8	14.5
B		✔			0.698	0.348	6.4	8.3
C			✔	✔	0.733	0.382	6.5	13.2
TDD-YOLO	✔	✔	✔	✔	0.775	0.415	7.1	20.5

✔ indicates the corresponding module is added.

**Table 16:** The 4G communication link performance metrics.

Metric	Average Value	Standard Deviation	Requirement for Real-Time Operation
End-to-End latency	91 ms	±24 ms	<200 ms
Packet loss rate	0.15%	±0.08%	<1%
Uplink bandwidth	17 Mbps	±4 Mbps	>5 Mbps
Downlink bandwidth	34 Mbps	±6 Mbps	>2 Mbps
TDD-YOLO End-to-End latency	28 FPS	±3 FPS	>24 FPS

**Table 17:** Class imbalance.

Model	Class	Precision	Recall	mAP@0.5	mAP@0.5:0.95
cls-loss = 0.5	All	0.886	0.801	0.873	0.508
	Improper tie	0.906	0.873	0.932	0.530
	Tension clamp miss	0.864	0.703	0.814	0.487
cls-loss = 0.75	All	0.889	0.814	0.892	0.511
	Improper tie	0.904	0.874	0.931	0.528
	Tension clamp miss	0.874	0.754	0.854	0.494

## Data Availability

The datasets used during the current study are available from the corresponding author upon reasonable request.

## Appendix A. Module Input and Output Details

**Table A1:** Model architecture input and output channels.

Layer	Module	Input [C,H,W]	Output [C,H,W]
0	Conv	[3, 640, 640]	[64, 640, 640]
1	space_to_depth	[64, 640, 640]	[256, 320, 320]
2	Conv	[256, 320, 320]	[128, 320, 320]
3	space_to_depth	[128, 320, 320]	[512, 160, 160]
4	C3k2	[512, 160, 160]	[256, 160, 160]
5	Conv	[256, 160, 160]	[256, 160, 160]
6	space_to_depth	[256, 160, 160]	[1024, 80, 80]
7	C3k2	[1024, 80, 80]	[512, 80, 80]
8	Conv	[512, 80, 80]	[512, 80, 80]
9	space_to_depth	[512, 80, 80]	[2048, 40, 40]
10	C3k2	[2048, 40, 40]	[512, 40, 40]
11	Conv	[512, 40, 40]	[1024, 40, 40]
12	space_to_depth	[1024, 40, 40]	[4096, 20, 20]
13	C3k2	[4096, 20, 20]	[1024, 20, 20]
14	CBAM	[1024, 20, 20]	[1024, 20, 20]
15	SPPF	[1024, 20, 20]	[1024, 20, 20]
16	Upsample	[1024, 20, 20]	[1024, 40, 40]
17	BiFPN_Concat	[1024, 40, 40] + [512, 40, 40]	[512, 40, 40]
18	C3k2	[512, 40, 40]	[512, 40, 40]
19	Upsample	[512, 40, 40]	[512, 80, 80]
20	BiFPN_Concat	[512, 80, 80] + [512, 80, 80]	[512, 80, 80]
21	C3k2	[512, 80, 80]	[256, 80, 80]
22	Upsample	[256, 80, 80]	[256, 160, 160]
23	BiFPN_Concat	[256, 160, 160] + [256, 160, 160]	[256, 160, 160]
24	C3k2	[256, 160, 160]	[128, 160, 160]
25	Conv	[128, 160, 160]	[256, 80, 80]
26	BiFPN_Concat	[256, 80, 80] + [256,80,80] + [256,80,80]	[256, 80, 80]
27	C3k2	[256, 80, 80]	[256, 80, 80]
28	Conv	[256, 80, 80]	[512, 40, 40]
29	BiFPN_Concat	[512, 40, 40] + [512,40,40] + [512,40,40]	[512, 40, 40]
30	C3k2	[512, 40, 40]	[512, 40, 40]
31	Conv	[512, 40, 40]	[512, 20, 20]
32	BiFPN_Concat	[512, 20, 20] + [1024, 20, 20]	[512, 20, 20]
33	C3k2	[512, 20, 20]	[1024, 20, 20]
34	Detect	[128, 160, 160] + [256, 80, 80] + [512, 40, 40] + [1024, 20, 20]	-
